# Effects of Three-Month Intake of Synbiotic on Inflammation and Body Composition in the Elderly: A Pilot Study

**DOI:** 10.3390/nu5041276

**Published:** 2013-04-17

**Authors:** João Valentini Neto, Camila Maria de Melo, Sandra Maria Lima Ribeiro

**Affiliations:** 1 School of Arts, Sciences and Humanities, University of São Paulo, São Paulo, CEP 03828-000, Brazil; E-Mail: joaovneto@gmail.com; 2 Federal University of São Paulo, São Paulo, CEP 04020-050, Brazil; E-Mail: cami_maria4@hotmail.com

**Keywords:** symbiotic, aging, inflammation, BIVA, body composition

## Abstract

We hypothesize that improvements in the gut microbiota are capable of ameliorating gut permeability and, consequently, reducing systemic inflammation and the risk of frailty. This study aims to evaluate some effects of synbiotic supplementation on inflammatory markers and the body composition of the elderly at risk of frailty. In a double-blind study that lasted three months, 17 elderly individuals fulfilling one frailty criteria (grip strength) were randomly distributed into two groups: SYN (*n* = 9), daily intake of synbiotic (6 g *Frutooligossacarides*, 10^8^ to 10^9^ CFU *Lactobacillus paracasei*, 10^8^ to 10^9^ CFU *Lactobacillus rhamnosus*, 10^8^ to 10^9^ CFU *Lactobacillus acidophilus* and 10^8^ to 10^9^ CFU *Bifidobacterium lactis*), or placebo (maltodextrin; PLA; *n* = 8). Subjects were analyzed for anthropometric measurements, bioelectric impedance with vectorial analysis (BIVA), IL-6 and TNF-α. A comparison between groups did not show any difference for the variables investigated. In turn, individual analysis of electrical impedance (BIVA) demonstrated that the majority of SYN individuals maintained or improved their tissue hydration, when compared to the PLA group after supplementation. In conclusion, three months of synbiotic supplementation did not promote any significant changes in inflammatory cytokines or body composition, but demonstrated a trend towards a preservation of hydration status in apparently healthy elderly individuals.

## 1. Introduction

Aging results in alterations in body functions, leading to consequences such as the reduction in fat-free mass (FFM). FFM, besides its structural role, is fundamental for the mobilization of metabolic substrates and for essential molecules synthesis, amongst other mechanisms [1,2]. In addition, skeletal muscle is the main source of body protein, and is capable of driving antibody production, wound healing and white blood cell production during acute or chronic diseases [3,4,5]. Therefore, if lean mass is depleted, there is less protein to support the body’s functionality, enhancing the risk of disabilities, reducing muscle power and/or physical function, increasing the risk of developing frailty syndrome [6]. Based on this evidence, investigations have tried to identify the origin of sarcopenia, a component of frailty syndrome, in order to prevent it or to reduce its consequences.

There are a number of physiological, metabolic, genetic and lifestyle factors that can favor the development of sarcopenia. Among these factors, low-grade systemic inflammation is considered to be important; low-grade inflammation associated with aging has been named, by some authors, as *inflammaging* [7], and is shown to have a complex integration with different body tissues/systems and somato senescence. Adipose tissue, gut and its microbiota have been implicated in the development of low-grade systemic inflammation in aging [8,9,10]. Changes in the gastrointestinal tract, as well as modifications in the diet and host immune system, affect the gut microbiota [8,9,10], for instance, increasing gram-negative bacteria (mainly *Enterobaceria*) and reducing *Lactobacilus* [9,11]. In turn, an imbalance in gut microbiota can modify gut permeability, which allows some of its content (bacteria and/or bacteria fragments, particularly the Gram-negative-derived lipopolysaccharides, LPSs) to pass through the gut wall towards the circulation, which is termed *metabolic endotoxemia* [12,13]. These molecules reach specific receptors (*i.e.*, *toll-like receptor*-TLR-4), in the adipose tissue and muscle tissue, among others, stimulating macrophage infiltration and activating the synthesis of inflammatory cytokines. This condition can contribute to outcomes such as reduced insulin sensitivity and sarcopenic obesity [8,9,12,14]. In skeletal muscle, cytokine signaling can result in enhanced catabolism and inhibit protein synthesis, modifying contractibility and muscle function as a consequence [9,15].

As such, we hypothesize that improvements in gut microbiota are capable of ameliorating gut permeability, therefore reducing systemic inflammation and its consequences. One strategy for improving the gut environment is the intake of prebiotic, probiotic or synbiotic substances, alone or in combination with food [16,17]. In brief, probiotics are “*live microorganisms which when administered in adequate amounts confer a health benefit on the host*” [18]. Prebiotics are “*nondigestible food ingredients that, when consumed in sufficient amounts, selectively stimulate the growth and/or activity of one or a limited number of microbes in the colon, resulting in documented health benefits*” [19]. A synbiotic is a combination of prebiotic and probiotic substances [20]. A number of studies have shown benefits of the intake of these substances in the management of diseases associated with systemic inflammation [21,22].

Considering that sarcopenia is an important manifestation of aging, with health and economic consequences, the present study aimed to evaluate the effects of the intake of a synbiotic substance on systemic low-grade inflammation and body composition in elderly individuals with a risk of frailty.

## 2. Experimental Section

### 2.1. Volunteer Recruitment and Ethical Aspects

The experiment included a convenience, non-probabilistic sample and was conducted from January to August 2010. All the volunteers participated simultaneously; the recruitment was made at January and February, the experiment was performed from March to May, and the results analysis was made from June to August. Volunteers were community-dwelling elderly, recruited from three different institutions and/or associations: an open university for older individuals, a recreational association and a physiotherapy center. The researchers extended a general invitation at these locations, and the subjects who were willing to take part in the experiment were checked for their eligibility. To be included, the participants had to be between 60 and 75 years old, free-living, and had to fulfill one of the frailty criteria proposed by Fried *et al.* [3]: the handgrip strength. As exclusion criteria, subjects should not report, at least three months prior the experiment, acute or chronic inflammatory gut diseases, use antibiotics, prebiotic, probiotic or synbiotic substances, or use of laxatives or anti-diarrhea medication. All the procedures were explained to the participants, who signed their informed consent. The project was approved by the local ethics committee (São Judas Tadeu University, COEP, São Paulo, SP, Brazil), protocol number 010/2010. 

### 2.2. Study Design

The volunteers were randomly distributed into two groups, in a double-blind experiment, lasting three months. One group (SYN) received one daily dose of a commercially available synbiotic substance, registered by the Brazilian Agency of Sanitary Surveillance-ANVISA (6 g *Frutooligossacarides*, 10^8^ to 10^9^ CFU *Lactobacillus paracasei*, 10^8^ to 10^9^ CFU* Lactobacillus rhamnosus*, 10^8^ to 10^9^ CFU *Lactobacillus acidophilus* and 10^8^ to 10^9^ CFU *Bifidobacterium lactis*). The PLA group ingested a placebo (maltodextrin). Randomization was performed by a colleague that was not taking a direct part in the study. Using a sheet with the names of the participants, the names were manually sorted out into groups, and from that time onward each were identified by a number. The same colleague organized the closed packages containing the powder to be ingested (placebo or synbiotics), labeling the packages with the numbers. The packages were similar for both groups, and the substances did not differ in color or flavor. The names of the subjects were revealed to the researchers only after the end of the experiments.

The subjects were instructed to dilute the powder in water and to drink the entire content of the resulting liquid following the last meal of the day. In addition, the participants were advised to not modify their daily habits; including meal times. To ensure that food and drink ingestion were not modified during the experiment; the participants were instructed to record daily food diaries; which were checked weekly during phone calls from the researchers. During these phone calls the researchers also checked whether the supplement was consumed correctly; and discussed any side effects.

### 2.3. Variables Investigated Before and after Three Months of Supplementation

Grip strength, which was one of the inclusion criteria, was evaluated with a dynamometer (Kratos Equipments^®^). The cutoff points to include the individuals in the study followed the recommendations of the American Society of Hand Therapists [23], which was proposed by Fried *et al.* [3]. Body mass (Filizola^®^ scale with 0.1 g precision) and height (Secca^®^ stadiometer to the nearest 0.1 cm) were evaluated to calculate the body mass index (BMI, kg/m^2^). In addition, skin fold measurements were taken from the triceps, subscapular, suprailiac, abdominal and calf (Lange^®^ caliper). Waist, hip and calf circumferences were also measured. The anthropometric procedures were based on Lohman, Roche & Martorell [24] and the measurements were always performed by the same experienced researcher.

Bioelectrical impedance analysis (BIA) was carried out after an overnight fast. BIA (Biodynamics 450e^®^) was performed while the participants were in a supine position and on a non-conductive surface, with the electrodes placed on the recommended locations on the hands and feet. The data were analyzed for resistance (R) and reactance (Xc), and plotted on an R/ht Xc/ht graph (vectorial analysis-BIVA). To plot the graphs, BIVA software was utilized (Department of Medical and Surgical Sciences, University of Padova, Padova, Italy, 2002) [25,26,27]. In addition, from the measurements of resistance and reactance, the fat mass and fat free mass were estimated, according to the equations provided by the equipment’s manufacturer [28].

Blood samples were collected after an overnight fast. After centrifugation, plasma was collected and stored for the analysis of IL-6 and TNF-α by ELISA (Diasource kits CAT# KP1261 sensibility 2 pg/mL and CAT#KAP1751, sensibility 0.7 pg/mL, respectively). 

### 2.4. Data Analysis

All the statistical analyses were conducted by the researchers, under the supervision of an experienced statistician. Levene’s test showed a normal distribution of all the variables investigated. Groups were compared by repeated measures ANOVA, followed by the LSD post-hoc test, using Statistica software, 11 (StatSoft, 1984-2012^®^). The BIA vector analysis was performed with BIVA software (Department of Medical and Surgical Sciences, University of Padova, Padova, Italy, 2002). An acceptable level of significance was established as *p* < 0.05 for all the analyses performed. 

## 3. Results

Forty two individuals volunteered for the study. Of these, 25 did not fulfill the inclusion criteria, and 18 of these attended the initial evaluation; one of these individuals interrupted the treatment, due to personal reasons not related to the side effects of the supplement. Therefore, 17 participants, of both genders (8 PLA (5 women and 3 men) and 9 SYN (8 women and one man)) concluded the study. During the final evaluation (post-supplementation), one subject, allocated to the PLA group, and one subject allocated to the SYN group did not perform the BIA analysis, due to technical problems. As such, for the BIA analysis, the sample was composed of 7 PLA and 8 SYN individuals. The mean age of the participants was 67.9 ± 4.5 years old (range 60–74 years).

Table 1 describes anthropometric, BIA and grip strength measurements. There were a small number of variables that presented significant differences (body mass, subscapular skin fold, suprailiac skin fold), between groups or between the time points.

**Figure 1:**
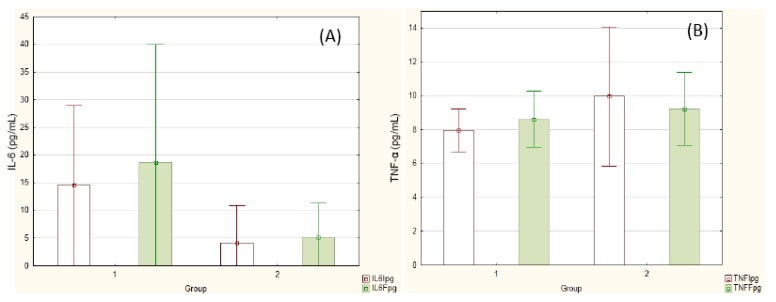
Cytokine concentration before (white columns) and after (green columns) supplementation. (**A**) IL-6; (**B**) TNF-α. Group 1 = PLA (*n* = 8); Group 2 = SYN (*n* = 9). Repeated measures ANOVA.

Figure 1 reports inflammatory cytokine concentrations before and after the experiment. The values did not differ either between groups or between the time points.

**Table 1:** Variables evaluated before and after the supplementation.

Variable	PLA		SYN	
	Initial (*n* = 8)	Final (*n* = 8)	Initial (*n* = 9)	Final (*n* = 9)
*Anthropometric measurements*				
BM (Kg)	69.6 ± 22.6 *	73.5 ± 22.6 *	64.9 ± 13.6	64.3 ± 13.7
BMI (Kg/m^2^)	28.5 ± 6.5	30.3 ± 6.4	27.9 ± 6.9	28.1 ± 7.1
TSF (mm)	21.0 ± 7.2	22.8 ± 4.4	17.3 ± 7.3	17.0 ± 7.1
SsSK (mm)	20.4 ± 4.2	22.5 ± 3.1	16.4 ± 6.5 *	17.9 ± 7.3 *
BSF (mm)	14.1 ± 7.0	16.4 ± 5.4	10.2 ± 5.5	10.3 ± 5.5
SiSF (mm)	19.7 ± 5.4 *	24.7 ± 3.4 *^,a^	15.4 ± 8.1	15.4 ± 9.2 ^a^
AbSF (mm)	26.0 ± 5.4	28.0 ± 3.6	21.9 ± 9.1	22.6 ± 8.6
CSF (mm)	21.9 ± 11.0	21.1 ± 8.9	17.1 ± 8.3	18.0 ± 8.3
WC (cm)	98.8 ± 18.3	100.5 ± 16.4	94.8 ± 15.7	93.3 ± 15.3
HC (cm)	101.6 ± 11.0	102.5 ± 8.7	101.4 ± 9.3	101.3 ± 9.7
CC (cm)	37.9 ± 5.3	39.0 ± 5.0	37.5 ± 6.1	37.4 ± 5.7
*BIA measurements*				
R/Ht (Ω/m)	358.3 ± 57.0	358.6 ± 79.8 ^##^	368.0 ± 73.4	377.3 ± 67.0 ^#^
Xc/Ht (Ω/m)	31.2 ± 9.7	34.7 ± 9.9 ^##^	32.7 ± 9.5	31.5 ± 7.7 ^#^
*BIA estimation of body composition*				
% BF	36.0 ± 7.0	38.6 ± 6.4 ^##^	35.6 ± 10.0	36.2 ± 10.7 ^#^
FM (Kg)	26.2 ± 13.3	29.3 ± 13.9 ^##^	23.9 ± 10.1	24.1 ± 10.5 ^#^
FFM (kg)	43.6 ± 10.6	44.4 ± 11.7 ^##^	41.0 ± 6.9	40.2 ± 6.5 ^#^
*Dinamometer measurements*				
Grip Strength (N)	15.9 ± 2.7	17.2 ± 3.9	15.0 ± 5.2	15.7 ± 5.3

^#^ *n* = 8; ^##^ *n* = 7; * significant difference between pre and post values (*p* < 0.05); ^a^, significant difference between placebo and synbiotic at post values (*p* < 0.05); ANOVA for repeated measures; TSF, triceps skinfold; SsSF, subscapular skinfold; BSF, biceps skinfold; SiSF, suprailiac skinfold; AbSF, abdominal skinfold; CSF, calf skinfold; WC, waist circumference; HC, hip circumference; CC, calf circumference; R/Ht, resistance divided by height; Xc/H, reactance divided by height; %BF, percentage of body fat; FM, fat mass; FFM, fat free mass.

**Figure 2:**
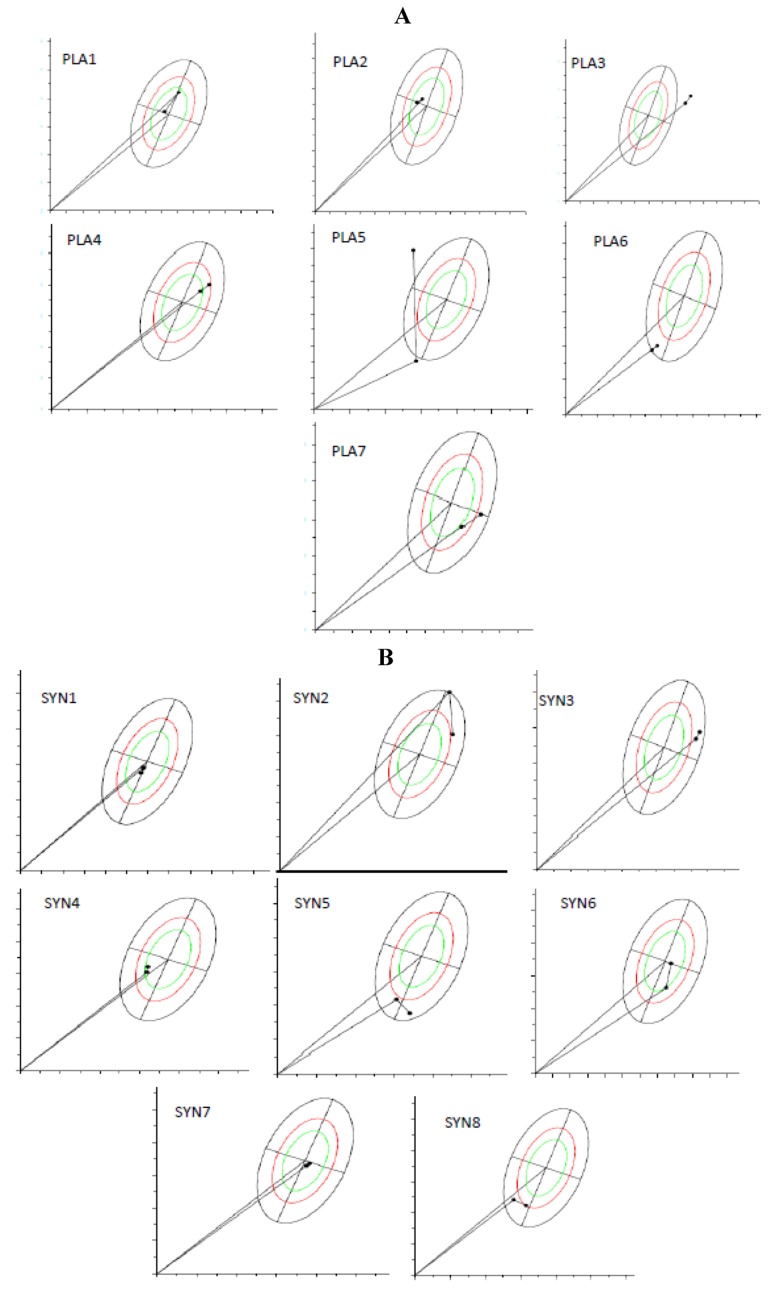
R/Xc path graph of vector migration. (**A**) PLA group (*n* = 7) and (**B**) SYN group (*n* = 8). The vectors were plotted on the reference 50th, 75th, and 95th tolerance ellipses of a reference healthy population. The path graph starts with an arrow from the origin ((0.0) point in the graph). When observing the arrow, the first point is the initial value and the second is the final value. To understand the meaning of vector migration, the major axis refers to hydration status (dehydrated individuals tending towards the upper pole). BIVA software (Department of Medical and Surgical Sciences, University of Padova, Padova, Italy, 2002).

Figure 2A,B shows the BIVA analysis from the RXc path graphs, which demonstrate vector migration, from before and after supplementation, for both groups. It is important to notice that the figures show individual vector migration. Therefore, this information does not demand statistical analysis of the group, and do not allow to point which group improved or which group jeopardized the hydration status. However, it is possible to highlight some information from these individual graphics. For the PLA group (A), 6 out of 7 participants (individuals 2–7, totaling 85.7% of the group) demonstrated a trend for vectors migrating towards dehydration (vectors migrated towards the upper pole), and therefore, only one individual (individual 1, totaling 14.3%) showed enhanced tissue hydration (vector migrated towards the bottom pole). For the SYN group (B), 5 out of 8 participants demonstrated vectors that pointed towards maintenance (individuals 7 and 8) or better hydration (individuals 1, 2 and 5), totaling 62.5%, and 3 individuals (individuals 3, 4, and 6, totaling 37.5%) demonstrated migration towards lower hydration. Summarizing, the majority of the individuals allocated in PLA group had their vectors toward dehydration, whilst the majority of the individuals at SYN group maintained or improved their hydration status.

## 4. Discussion

We hypothesized that synbiotic supplementation could present benefits to the elderly, keeping in mind the following sequence of events: improvement in gut microbiota, reduction in systemic inflammation, and slowdown of fat-free mass reduction. Our randomized study, considering our experimental conditions, did not suggest any expressive advantage for synbiotic supplementation. The great majority of the data analyzed did not show significant differences, either between groups or between time points.

Despite the lack of significance between groups for the majority of the analyses, individual BIVA results suggested some trends. The majority of the volunteers allocated to the PLA group demonstrated, at the end of the experiment, a migration of their vectors towards dehydration, and this fact was not observed in the majority of individuals allocated to the SYN group. These very subtle trends could indicate, in the PLA group, some water imbalance. It is important to remember that we analyzed the inflammatory cytokine concentration, together with BIVA (an indirect investigation of tissue hydration); during inflammation, variables associated with capillary permeability are modified, causing changes in colloid-osmotic pressure as well as in plasma and interstitial fluid pressure. These changes result in a water imbalance [29], and could interfere in processes such as protein synthesis [29,30,31]. Therefore, protein synthesis (and consequently the improvement and/or maintenance of muscle mass) starts with a good water balance in the muscle fiber environment [32].

It is interesting to note that the PLA group, besides presenting a trend towards lower hydration, demonstrated an enhancement in body weight and suprailiac skin fold. We may assume from these data, that the PLA group presented a slightly higher adiposity. It is known that the higher the adiposity, the lower the hydration [33], and therefore we can hypothesize that as SYN supplementation maintained the hydration status, it impaired or reduced fat gain. We must remember that fat gain is very common during the aging process, and is one of the factors associated with sarcopenic obesity [31]. However, it is important to bear in mind that anthropometric measurements have to be analyzed as a whole, and most of the measurements made did not present significant differences. Additionally, the concentration of inflammatory cytokines did not support our hypothesis (considering the sequence: gut microbiota, gut permeability, inflammation and muscle mass).

Probably one difficulty in finding differences between groups in the present study was the recruitment of supposedly healthy (free-living) elderly. It is important to remember that during the aging process, even in non-pathological situations, a reduction in body water (and therefore in FFM) has been observed both in men and women [32]. These reductions are expected to be very slow and subtle and may justify the absence of significant differences in our study. In fact, for this reason, we investigated the “reduction in losses”, rather than “enhancement” in fat-free mass. As such, we can infer that a longer time of supplementation may be necessary. Additionally, for further studies, considering the hypothesis that the gut is one of the main sites responsible for the beginning of low-grade inflammation in the elderly, the measurement of variables associated with the gut permeability may be more sensitive to the possible effects of synbiotic supplementation (for instance, LPS). Therefore, the main limitations of our study are: the small sample size (which is a very frequent problem in studies with the elderly), the short time for supplementation and the lack of more specific markers of gut permeability. It is important to highlight that we cannot generalize our results, principally due to the existence of individual, regional or national differences in gut microbiota. 

## 5. Conclusion

According to our experimental conditions, three months of synbiotic supplementation did not promote significant differences in variables related to inflammation or body composition in the elderly. However, we observed a subtle trend towards an improved hydration status, which could signify, in the long term, benefits for the FFM. Sarcopenic processes should be monitored, due to their relationship with outcomes such as frailty and, therefore, further experiments of longer duration, with the measurement of specific markers of gut permeability and inflammation, should clarify our hypotheses. We believe that systemic inflammation reductions, as well as changes in fat free mass, demand longer than three months to be identified from biomarkers such as cytokines, LPS or even body composition method. 
